# The Implementation of a Primary HPV Self-Testing Cervical Screening Program in Malaysia through Program ROSE—Lessons Learnt and Moving Forward

**DOI:** 10.3390/curroncol29100579

**Published:** 2022-10-02

**Authors:** Yin Ling Woo, Su Pei Khoo, Patti Gravitt, David Hawkes, Reena Rajasuriar, Marion Saville

**Affiliations:** 1 Faculty of Medicine, University of Malaya, Kuala Lumpur 59100, Malaysia; 2 ROSE Foundation, Kuala Lumpur 50603, Malaysia; 3 Centre of Global Health, National Institute of Health, Rockville, MD 20892, USA; 4 Australia Centre for the Prevention of Cervical Cancer (ACPCC), Victoria 3053, Australia; 5 Centre of Excellence for Research in AIDS, University of Malaya, Kuala Lumpur 59990, Malaysia

**Keywords:** self-sampling, cervical screening, HPV testing, cervical cancer elimination

## Abstract

Program ROSE (removing obstacles to cervical screening) is a primary HPV-based cervical screening program that incorporates self-sampling and digital technology, ensuring that women are linked to care. It was developed based on the principles of design thinking in the context of Malaysia. The program illustrates the importance of collaborative partnerships and addressing the multi-faceted barriers from policy changes, and infrastructure readiness to the implementation of a radically new cervical screening program in communities. The paradigm shift in cervical cancer requires a monumental and concerted effort in educating both the healthcare providers and the general public. In this short review, we highlight how Pilot Project ROSE incorporated evidence-based tools that rapidly scaled up to Program ROSE. These ideas and solutions can be adapted and adopted by other countries. Notwithstanding the impact of COVID-19, it is incumbent on countries to pave the road towards the elimination of cervical cancer with pre-existing footpaths.

## 1. Tailoring the Global Roadmap towards Cervical Cancer Elimination to the Real-World Setting

Cervical cancer is a disease of inequity, with 85% of cases and deaths occurring in low and middle-income countries [1]. In 2019, the World Health Organization (WHO) made a call towards the elimination of cervical cancer as a public health problem by vaccinating 90% of female adolescents, screening 70% of eligible women with a high-performance test (HPV test) at least twice a lifetime and ensuring 90% of women who are screened positive will receive appropriate treatment [2]. It is estimated that the rapid scale-up of these interventions would result in most countries achieving the elimination goal of new cases of cervical cancer at less than 4 per 100,000 women by 2100 [3]. As of 2021, considerable disparities in the uptake of HPV vaccination are observed between countries; with more than 85% of high-income countries having introduced a national HPV immunization program while less than 25%, in lower-income countries. This is coupled with the lack of a national cervical cancer screening program and limited facilities available for diagnosis and treatment in the public sector of many lower-income countries [4]. Therefore, it will be incumbent on countries to pave the road to the elimination from pre-existing footpaths. Notwithstanding the impact of COVID-19 from which the world is recovering, many countries are experiencing the devastating effects of climate and humanitarian crises [5]. As an example, at the end of 2021, The UN Refugee Agency (UNHCR) reported that 89.3 million people worldwide were forcibly displaced because of persecution, conflict, human rights violations or events seriously disturbing public order [6]. Hunger has become a direct threat to the health and survival of millions [7]. In June 2022, the World Food Program (WFP) reported that 750,000 people were at immediate risk of ‘starvation or death’ with 276 million people facing acute food insecurity [8]. These human emergencies are real and catastrophically impact women who are already at the highest risk of cervical cancer. The progress made over the years in healthcare has lost its ground in many countries.

It is against this very background that we need to consider how each country can support and work towards the 90–70–90 targets for cervical cancer elimination. In the long run, the cost of inaction will catch up resulting in more loss of women’s lives.

## 2. The Malaysian Landscape

In Malaysia, cervical cancer is still the third most common and the fourth most deadly cancer among women with an age-standardized incidence rate (ASIR) of 10.2 per 100,000 women, representing 1740 new cases diagnosed in 2020 [1]. The number of new cases and deaths is estimated to rise by 64% and 87% in 2040, respectively, if no immediate action is taken [9]. As an upper-middle-income country, cancer diagnosis and treatment services such as pathology, radiotherapy, chemotherapy and surgery are available in Malaysia through a mixed public-private health care delivery system [10,11].

In 2010, Malaysia introduced a national HPV vaccination program using a school-delivery model, vaccinating the single-age female cohort at the age of 13 [12] with a high coverage of >80% in the past 10 years [13,14] However, the national HPV vaccine delivery program has been significantly affected since the COVID-19 pandemic. In 2020 and 2021, more than 200,000 school-going-13-year-old girls would have either missed their vaccination or have incomplete doses due to school closures [15,16,17] or disruption of HPV vaccine supply chain [17].

In terms of cervical screening, the conventional Pap smear has been available in Malaysia since the 1960s and was expanded nationwide through the launch of the national screening program in 1998, targeting all eligible women aged 20–65 years old [18]. It is an opportunistic program where Pap smear are offered to women who attend primary care clinics [19,20]. There is also a lack of national screening registry, thus information related to follow-up care is not available. The Ministry of Health accommodates a large portion of cervical screening services (75%) at no cost to the public and the remaining are provided by other agencies such as the Ministry of Women, Family and Community Development, university hospitals, private facilities, and non-governmental organizations [21,22,23]. Despite wide availability, the screening uptake has been poor, at about 25% in the past decades; and this has reduced by at least 50% since the COVID-19 pandemic [16].

Malaysia is committed to achieving cervical cancer elimination. The Ministry of Health now fully endorses HPV tests via self-collection method as the primary cervical screening method in phases by 2023/2024 [24,25]. This exists within the larger Action Plan Towards Elimination of Cervical Cancer in Malaysia (2021–2030) which encompasses elimination goals and targets for the scale-up of vaccination, cervical cancer screening and treatment [11].

## 3. Overcoming Barriers to Implementing the WHO Cervical Cancer Elimination Targets

### 3.1. Political Commitment and Costs Justification

It is important that national cancer screening programs are evidence-based and cost-effective. There is an overwhelming body of evidence to demonstrate that screening programs based on HPV testing are more effective than Pap-based programs [26]. These findings have underpinned WHO guidelines for screening and treatment of pre-cancer lesions for cervical cancer prevention [27]. WHO recommends that member states move towards offering HPV-based primary screening every 5 to 10 years. Furthermore, HPV-based primary screening has also been shown to be cost-effective in all settings, including LMICs [28].

In this context, the decision to allocate the necessary resources is ultimately political and arguably a reflection of how governments value the lives and the health of their women, particularly those from disadvantaged populations. One of the biggest hurdles in adopting an HPV-based screening program is getting it approved at government level because of the perceived high financial costs. Too often policy makers assess the cost of the HPV test and decide that the country cannot afford this investment.

In contrast, HPV-based screening using well-established and clinically validated assays can be highly automated with excellent protection afforded with 5-to-10-year screening intervals. Indeed, even two tests in a lifetime, provided these are spaced by at least 10 years, will have significant impact on cervical cancer incidence and mortality [29,30].

### 3.2. Infrastructure and Healthcare Providers for Cervical Screening

The usual providers for cervical screening services include community health workers, nurses, primary care doctors, gynaecologists, gynaecological oncologists, pathologists, and cytopathologists to name a few, depending on how each country delivers its existing cervical screening program. The system in which these different providers work in is usually embedded within a public or private healthcare setting or an NGO-based health facility with ‘fixed’ perspectives on the appropriate screening and follow-up method. While these beliefs may be based on good clinical practice, they are often not considered with an equity lens, where resources and circumstances may preclude a large sector of the population from accessing these ‘best practices’.

Population primary HPV tests on the other hand can be scaled up very quickly. High-capacity PCR platforms can either be purchased outright, incurring significant capital costs, or they can be negotiated in conjunction with the purchase of test consumables. A silver lining coming out of the COVID pandemic is that many countries procured and installed high-capacity PCR platforms for SARS-CoV-2 testing. As demand for SARS-CoV-2 testing declines, there is an opportunity for countries to explore whether they can leverage this capacity to facilitate HPV testing.

Hence, one of the most challenging aspects of implementing a paradigm shift in cervical screening is to ensure that providers are convinced that HPV testing is superior to conventional cytology as a screening tool and has added advantage in affording an option for self-collection of the screening sample. By not requiring a speculum exam for the primary screen, health care providers can screen more women per day, and women can be reached conveniently through community health campaigns.

### 3.3. Knowledge and Acceptance of Cervical Screening

While the biggest obstacle to screening is having access to the screening tools, what is becoming clear is this: women have not come forward to be screened even when it is accessible. Whether it is the fear of the unknown or that health screens are not relevant to them, many in fact do not access preventive health measures. Unfortunately, we still have big gaps in understanding women’s health-seeking behaviour from different geographical and socio-cultural backgrounds when it comes to prevention. In the setting of an LMIC such as Malaysia, women do not attend regular screening for a variety of reasons such as fear, embarrassment, inconvenience and lack of awareness [31]. Health systems as in Malaysia which have for decades underinvested in primary prevention will face additional challenges in raising awareness and changing the mindset of women on the importance of prevention vs. treatment. Additionally, efforts will have to be made to aggressively address the myths and stigma associated with HPV testing [32].

## 4. The Evolution of Program ROSE (Removing Obstacles to Cervical Screening)

To address many of the barriers highlighted above, a radically different approach was required to increase the uptake of cervical screening. The tools that we have for the elimination of cervical cancer are evidence-based and readily available [27,33]. While transitioning to HPV-based screening programs, there tends to be disproportionately more attention given to the technical details such as HPV testing platforms or self-collecting devices. An important lesson gleaned from designing and executing Program ROSE is appreciating the importance of ‘program implementation’ from the perspective of the users (healthcare professionals and the women themselves).

### 4.1. Accelerating Innovation through Collaboration

**Figure 1:**
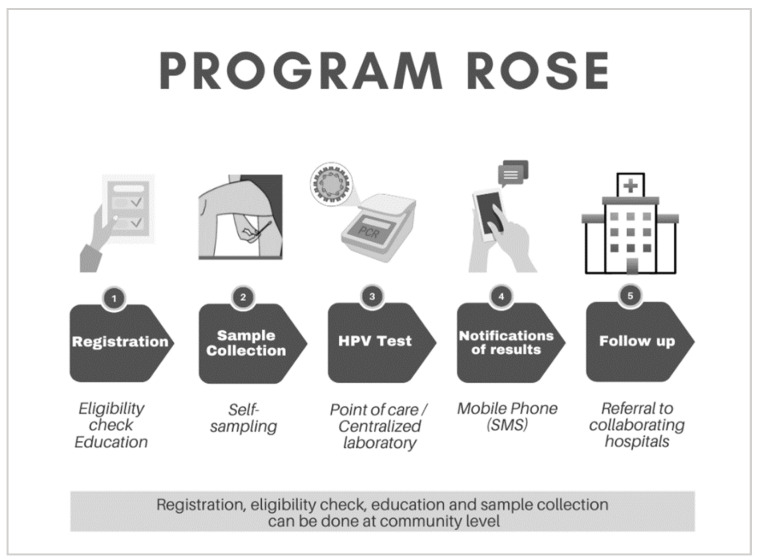
Overview of Program ROSE. Step 1–2 can be conducted in different settings such as primary healthcare facilities or in the community: Eligibility check, education and self-sample collection. Step 3: HPV testing can be point of care or in a centralized laboratory. Step 4: Results are delivered via SMS within a pre-determined time frame and Step 5: Participants with a positive screen will be supported by a dedicated program staff and linked to care.

Pilot Project ROSE started as a collaboration between the University of Malaya (UM), the Australian Centre for Prevention of Cervical Cancer (ACPCC), and independent experts. At its core, Pilot Project ROSE (later known as Program ROSE) integrates self-sampling, primary HPV testing and a digital registry, ensuring women who require follow-up are linked to care (Figure 1). Broadly, the UM team crowdsourced the resources required, contributed to the clinical care, and provided deep insights into the local communities while ACPCC brought technical support by way of laboratory and digital health expertise. As the pilot developed, collaborative partnerships became broader and included academics, implementors, institutions and individuals who shared the vision of making cervical cancer a rare disease in Malaysia.

The strategic roadmap provided by WHO was central in communicating the science and purpose behind the program to the public and policy makers. While many of the collaborator’s core expertise was not in healthcare, they embraced some level of uncertainty but believed that the goal was achievable. Throughout the process of designing Pilot Project ROSE, timely two-way communication was critical in building trust between all parties. Simultaneous discussions with telecommunication companies (TELCOs), computer programmers, legal departments, financiers, donors, doctors (gynaecologists, pathologists, primary care physicians), nurses, academics, policy makers and volunteers required a ‘different language’ to coordinate and convey the requirements for execution.

While it was important to have a bird’s eye view of the challenges faced with the current cervical screening program, a more detailed understanding of how the program was being delivered to the women in the primary care setting was vital. Stakeholder engagements were performed to explore the barriers from the implementor’s perspective (meetings and discussions with the ministry of health officers) and visits were made to the ministry of health primary care clinics (klinik kesihatan) to gain the perspectives of the healthcare providers and patients [34]. Structured interviews carried out among the different groups of health care providers (receptionists, nurses, doctors, attendants, and laboratory assistants in the clinics) provided an insight into the challenges encountered on a day-to-day basis. Moreover, the team explored how ready they were to accept a new modality of screening, in particular the introduction of self-sampling. Studies were simultaneously conducted to understand attitudes and acceptance of self-sampling among multi-ethnic Malaysian women [35]. The team sought a solution that would not disrupt the daily processes in these primary clinics that could be serving up to 1000 patients per day and to utilize existing resources (no need for new computers, printers, rooms or staffing), aligning it to the processes of the clinics and maximizing efficiency.

One of the initial benefits of the collaboration was getting the support of the manufacturers of HPV tests, and the related instrumentation. The budgets of many pilot studies are often diverted to the costs of the HPV tests (and instrumentation) and as such the most important features of a good program, such as engagement with the community, are conducted with minimal, or insufficient funding. The collaboration with a high-volume pathology laboratory (ACPCC) led to conversations with the HPV test manufacturers and the result was over 4000 tests donated by two different manufacturers (Cepheid for the point-of-care model and Roche for the centralized laboratory model). Furthermore, experts from our partner laboratory also provided subsequent technical support in relation to HPV testing. For instance, when approached by different manufacturers or their distributors with potentially new products, the team at ROSE have rapid access to experts for an independent and evidence-based perspective.

In February 2020, just as the COVID-19 pandemic was hitting, the ROSE Foundation Laboratory was officially launched. The partnership between the ROSE Foundation Laboratory and the collaborating pathology service continues today with clinical governance and scientific and technical support.

### 4.2. Acceptability of Self-Testing for HPV Testing in Malaysia

Organized population screening with conventional cytology in high-income countries has led to a reduction in cervical cancer mortality rates [36,37]. However, despite efforts to implement such costly cervical screening programs in less developed settings, these were met with limited success [38]. With the discovery that high-risk HPV is the necessary cause of cervical cancer [39], the scientific community consolidated their knowledge to develop effective vaccines and screening tools within a relatively short time, enabling the realization of eliminating a human cancer within the century [3]. The rapid development of HPV testing platforms that are validated for population screening supplemented by the ease of obtaining a sample through self-testing has nothing been short of revolutionary [40,41].

One of the biggest advantages of advocating for HPV testing as the primary screen is that a woman needs to only perform as few as two tests per lifetime with the option of self-testing. In the pilot phase, a survey of 1000 women found that more than 97% of women found the test acceptable and 99% would use this strategy rather than a physician-acquired sample. This information dispelled the myth that Malaysian women could not take their own samples and that it would lead to high invalid tests. In a recent Malaysian study, more than 80% of the participants perceived the self-sampling method as easy, convenient, not embarrassing, and confident in performing the test, indicating that self-sampling is highly acceptable. It was also the preferred choice of cervical screening compared to Pap smear and physician-sampling HPV test [31]. Over time the key messages to anyone asking about HPV testing became simple: Clinically validated, good support, and PCR-based for self-collection [41,42,43,44].

Program ROSE utilizes the COPAN 552C FLOQSwab® (COPAN Diagnostics Inc., Murrieta, CA, USA) for self-sampling. One of the major advantages is that these swabs can be stored in room temperature for up to 14 days before it is tested. Operationally, this means that community outreach programs can be conducted in rural or distant sites and only tested in the ROSE laboratory when the team returns with the swabs. The ease of transportation is a huge advantage particularly when community screens are not so accessible.

### 4.3. Success Measured by Linkage to Care

Any screening test without the appropriate follow-up does not save lives and is a waste of resources. One of the shortcomings in the Malaysian cervical screening program is the absence of information with regard to women requiring follow-up care. The norm for most women is the unstated rule that ‘no news is good news’.

Linkage to care was an important element built into Program ROSE with staff dedicated to ensuring women testing positive receive their results and are linked to care. This process is facilitated by a simplified algorithm that allows program staff, most of whom are non-medically trained, to engage with women, linking them to the nearest treatment centre. We have learnt this is a critical step in facilitating linkage to care as many women expressed anxiety and fear of cancer following receipt of a positive test result but responded well when reassured by program staff.

**Figure 2:**
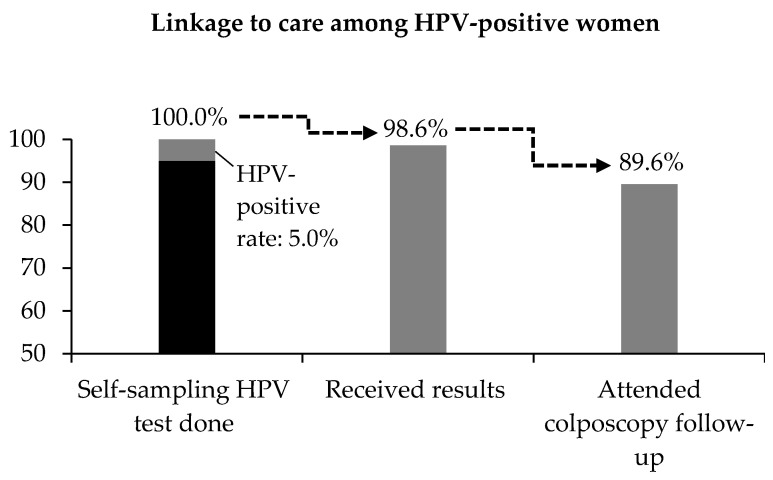
Linkage to care among women who were screened positive for self-sampling HPV test under Pilot Project ROSE. A total of 4188 women were screened and 99.7% had a valid HPV test result. The positive rate was 5.0%. Almost 99% of HPV-positive women confirmed receipt of test results and 89.6% attended colposcopy follow-up.

For Pilot Project ROSE, the analysis of about 4000 screens found this effort translated to 90% of women with positive tests attending follow-up (refer to Figure 2). Program ROSE has demonstrated that is possible to empower communities and train them to take on certain tasks with the support of professionals, thereby turning clinicians from being the do-ers to enablers. Women no longer need to attend their cervical screening in clinics or hospitals, relieving the stress in these facilities.

This linkage to care has been facilitated by the use of a digital registry powered by canSCREEN^®^ (Australia); a digital health solution that securely records the participants identifying and contact information, and results of tests and follow-up visits, giving the ROSE team a clear view of participants that are overdue for follow-up care so that they can be contacted.

## 5. Looking Forward and Concluding Remarks

Program ROSE in its design is agile and can adapt to demands made by unexpected circumstances. For example, at the peak of the COVID-19 pandemic, when large community gatherings were discouraged, Program ROSE carried out education and interacted with small groups of women by teleconference. One-to-one registration was done online, and the swabs were dropped off at strategic sites for collection. As the pandemic subsided, community screenings resumed with physical distancing and masking in well-ventilated spaces.

Continuous advocacy, engagement, and education with not just facts but active promotion is necessary to create awareness and demand which will then drive down the costs of tests, making HPV tests more feasible in low-income settings. One of the challenges with the WHO elimination strategy is the availability of cheap, clinically validated, PCR-based HPV tests which can handle the volume of a national cervical screening program. It has been proposed that a test with an all-inclusive price of under USD 5 would be needed to facilitate widespread HPV-based screening in LMICs, and none of the currently available tests are close to this target price [45].

Program ROSE has now screened more than 20,000 women in Malaysia, with high patient and healthcare worker acceptability, and proper linkage to care. It serves as a model for delivering high-quality screening services to those who might otherwise not be screened, and which could be scaled up nationally by Malaysia and other LMICs to reach the WHO screening (70%) and treatment (90%) targets.

Together with Malaysia’s longstanding and highly successful HPV vaccination program, perhaps Malaysia could be the first country in the region to reach the cervical cancer elimination target (4 per 100,000) by 2100 [3].
